# State Hospitals: Why English Men and Women Can Never Suffer Them

**Published:** 1914-01-10

**Authors:** 


### STATE HOSPITALS.

### Why English Men and Women Can Never Suffer Them.

### IMPORTANT SPEECH BY LORD NORTHCLIFFE AT COVENTRY.

Lord Northcliffe, who twenty-seven years ago was resident at Coventry in association with Mr. Iliffe, the father of the present chairman of the Coventry and Warwickshire Hospital, made an important speech at the annual meeting of this hospital on the 30th ultimo. We have long known that Lord Northcliffe was considering and weighing the hospital question, and that he had displayed considerable interest in the study of the voluntary hospital system in this country. His speech at Coventry possesses special importance from the fact that, it contains his matured views upon a State hospital system, based upon a personal inspection of some of the most famous and largest of this type of hospital. We take the following report of Lord Northcliffe's words from the *Midland Daily Telegraph* of the 31st ultimo. We could wish that what follows could, through the courtesy of the editors of British newspapers, find a place in each of our newspapers, daily and weekly.

### What Lord Northcliffe has Found.

I have always taken a particular interest in hospitals. Having, as some of you know, some 30,000 workpeople of my own, it has been necessary for me to build more than one hospital. So that when I came to see your magnificent enlarged hospital to-day I did not come as a mere tyro to see something which I did not understand. I have had the very interesting task of inspecting two of the greatest hospitals in Germany, the great hospital at Hamburg, and the Rudolf Virchow Hospital in Berlin. I have seen the great Victoria Hospital in Montreal—by many people accounted the finest in the world. I have seen most of the best New York hospitals, and nearly all our own London hospitals. I saw them with a view to learning something of the building and the management of hospitals, and I say it in no sense of flattery when I remark what I believe, that the extended part of your hospital has been designed by some architect with great skill and great experience.

### The Voluntary System.

The Mayor spoke of State hospitals and private hospitals. I am one of those old-fashioned people who believe in the English system of hospital management. I have travelled on State railways and I have been in State hospitals. We have abundant evidence of the way things are managed by the State in this country in watching the State manage telephones; other State departments I will not mention. What I have noticed about State hospitals, despite the great skill—especially which I saw in the Virchow Hospital of Berlin—of the professors of the system, is the apathy of the people working it. Our system of voluntary hospitals has produced beyond question the best hospital nurses in the world. That means throughout the world. Our system of voluntary hospitals produced Lord Lister, who revolutionised the voluntary hospitals of the world and the State hospitals of the world. I think those are two great factors in favour of our present system of hospital management.

### "A Cold, Soulless Business Machine."

When I went to Berlin to investigate hospitals, my German friends said, "Why do you want to see hospitals? Nobody here wants to see hospitals except those managing hospitals." I said, "In England a great many people take an enormous interest in hospitals who have no connection whatever with them." I found that the enormous national interest which we take in hospitals is unknown where hospitals are run as State departments. A person when he has paid so much feels that he has done his duty and there is an end of it, and the management of the hospital is left entirely in the hands of a non-criticised staff—in Germany of splendid scientific men—and the whole of the skill of the hospital lies in the design of the hospital, from which we learn a great deal, and in the medical administration. For the rest, the thing is a cold, soulless, business machine—which you cannot say of your hospital as I saw it to-day. There is nothing more touching, nothing more beautiful, to be seen in any city than the children's ward of the hospital as I saw it this afternoon, showing not a mere interest in a number of taxpayers, but all sorts of small affectionate signs of the citizens of Coventry's interest in their hospital.

### Sir Henry Burdett's Opinion.

Sir Henry Burdett, like Lord Northcliffe, has been specially to Germany, the United States, Canada, and other foreign countries. He has made an inspection of many of the principal State and municipal hospitals in various parts of the world. His reports on German hospitals have been republished from The Hospital in pamphlet form.

Sir Henry reports: "When the fullest recognition has been given to the relatively high technical efficiency of the best hospitals of Germany at present, we are bound to testify that English men and women would never rest content to be patients in the general wards of institutions, under an iron discipline, which deprives the patients in a hospital of the safeguards of an independent and articulate public opinion, and ministers to the setting back of humanity and the infinite lessening of the humanising influences, without which the spirit of personal service and trained nurses cannot be maintained, nor the delightful atmosphere which is to be found to-day in the best-administered of our voluntary hospitals throughout Great Britain. It follows that the more knowledge the people of these islands gain of a patient's life in municipal hospitals conducted on the foreign principle, where scientific treatment is apt to control affairs completely, the more certain it is that a great system of State and municipal hospitals will never be set up in Great Britain, to the exclusion or destruction of the voluntary hospitals, which confer such infinite blessings upon all classes at the present time."

